# In Praise of Calendula

**Published:** 1889-07

**Authors:** Alice B. Campbell


### IN PRAISE OF CALENDULA.

[I. H. A., June, 1889.]

On the night of July 4th, 1887, while riding on the rear platform of a car, on his way home from Astoria to Brooklyn, Chas. L., twenty-three years old, felt a trickling on his cheek, which on wiping he found to be blood. This was the first intimation he had that he had been shot in the right eye by a fellow-passenger who had been amusing himself during the trip by the repeated firing of a pistol. Charles L. was so frightened by the discovery—thinking if he had been shot he must surely die—that he fainted. He was taken to the police station, where his wound was dressed, then his home, meanwhile he had recovered consciousness.

I saw him the following morning about nine o'clock ; he was suffering great pain in the eye, and was unable to sleep. I removed the dressings which were of the popular antiseptic order. Cleaned the wound of the iodoform powder and carbolic mixture. The part wounded had lost all resemblance to an eye, the eyeball being completely obscured by a mass of mangled, swollen conjunctiva which protruded so as to hide the lids, making the whole look like a lump of raw meat.

After applying diluted Calendula, and giving Arnica internally—the effect of the shock seeming to indicate it—I found him next day free from pain, and more quiet mentally. Continued the treatment minus the Arnica.

Next day the swelling was greatly reduced, no suffering, and only complaining of sleepiness, with inability to sleep, which a dose of Belladonna removed entirely. I made no more visits, gave no more medicine, only continuing the application of Calendula.

The patient saw me on the sixth day. Said he was perfectly well, and free from pain, but could not see out of the wounded eye. The eye was restored to the normal size, showing a dull surface over whole eyeball, the wound perfectly healed, but a horizontal line about a quarter of an inch long, was perceptible outside the iris. I sent him to the New York Ophthalmic Hospital, where the eye was removed on the eighth day from the accident. In the orbit was found a ball of 38-calibre. In three weeks from time of accident the patient returned to his work, and has suffered no inconvenience since.

The Calendula was continued at the hospital after removing the eye, and there can be no doubt that to its specific action on mangled and torn surfaces is due the freedom from pain, the rapid healing and quick recovery which characterized this case both before and after the ball was extracted.

ALICE B. CAMPBELL.

